# Impact of Substituting Meats with Plant-Based Analogues on Health-Related Markers: A Systematic Review of Human Intervention Studies

**DOI:** 10.3390/nu16152498

**Published:** 2024-07-31

**Authors:** Cristian Del Bo’, Lara Chehade, Massimiliano Tucci, Federica Canclini, Patrizia Riso, Daniela Martini

**Affiliations:** Department of Food, Environmental and Nutritional Sciences (DeFENS), Division of Human Nutrition, Università degli Studi di Milano, 20133 Milano, Italylara.chehade@unimi.it (L.C.); federica.canclini@studenti.unimi.it (F.C.); daniela.martini@unimi.it (D.M.)

**Keywords:** plant-based foods, meat alternatives, sustainable healthy diets, dietary intervention

## Abstract

The growing drive towards more sustainable dietary patterns has led to an increased demand for and availability of plant-based meat analogues (PBMAs). This systematic review aims to summarize the currently available evidence from human intervention studies investigating the impact of substituting animal meat (AM) with PBMAs in adults. A total of 19 studies were included. Overall, an increase in satiety following PBMA intake was reported, albeit to different extents and not always accompanied by changes in leptin and ghrelin. PBMAs generally resulted in lower protein bioavailability and a smaller increase in plasma essential amino acids in comparison to AM. However, muscle protein synthesis and physical performance were not affected. Finally, conflicting results have been reported for other outcomes, such as pancreatic and gastrointestinal hormones, oxidative stress and inflammation, vascular function, and microbiota composition. In conclusion, we documented that the impact of substituting AM with PBMA products has been scarcely investigated. In addition, the heterogeneity found in terms of study design, population, outcomes, and findings suggests the need for additional high-quality intervention trials, particularly long-term ones, to better clarify the advantages and potential critical issues of such substitutions within sustainable healthy diets.

## 1. Introduction

A large body of evidence demonstrates that inadequate dietary habits are associated with detrimental effects on human health, especially in terms of increasing the risk of non-communicable diseases, such as cardiovascular diseases, diabetes, and certain types of cancers. For instance, results from the Global Burden of Disease showed that a diet low in foods to be promoted, such as fruit, vegetables, and legumes, and a diet high in salt, red meat, and processed meat are among the leading causes of mortality and disability-adjusted life-years [1]. At the same time, it is also well recognized that food systems impact planetary health. This impact includes water use, land use, and greenhouse gas emissions (GHGEs). In particular, evidence shows that animal-based foods are generally associated with a high environmental impact, especially when data are expressed per 100 g of weight without considering portion size, frequency of consumption, nutrient density, and bioavailability of nutrients [2].

Due to the increasing concern for human and planetary health, there is a growing awareness of the need to transition towards plant-based diets with reduced consumption of animal-based foods. For this purpose, several dietary patterns that consider the different aspects of sustainability and can promote the shift towards sustainable, healthy diets have been developed in recent years [3,4]. This transition is essential to reduce the environmental impact of food systems while simultaneously improving human health and animal welfare [5]. Among the several animal-based foods, meat is a subject of large debate for its impact on human and planetary health. On the one hand, meat is an important source of several nutrients, such as high biological value protein, heme iron, zinc, and vitamin B_12_ [6,7,8]. On the other hand, the high consumption of preserved and red meat has been considered a potential cause of an increase in non-communicable diseases [9,10]. Moreover, meat, especially from ruminants, has a higher environmental impact compared to other animal-based foods and plant-based foods [11,12]. Thus, there is interest in finding strategies to reduce the consumption of meat and to understand the impact of this reduction on human and planetary health [13,14,15,16].

Meat consumption can be reduced in various ways, such as reducing meat portion sizes, replacing meat with other protein sources, or applying the “less but better” principle, which aims to reduce quantity while increasing quality [17]. Additionally, in recent years, there has been an increasing trend in consuming plant-based meat analogues (PBMAs) that aim to mimic meat closely in terms of sensory and nutritional aspects, and even in product names [18,19]. For these reasons, the market is promoting more PBMAs, which have seen a remarkable increase in product launches in recent years [20]. These products vary widely with respect to sensory characteristics and ingredients used. Soy and wheat are generally used as the main sources of proteins, although the use of new sources, such as beans and pseudo-cereals, is increasing [21]. In regard to nutrient composition, despite their large variability, PBMAs generally have lower saturated fat and higher fiber content. However, they can also be naturally lower in micronutrients than animal meat (AM), such as vitamin B_12_ and iron, unless they are fortified [22,23,24]. Moreover, there is still concern about the actual bioavailability of these micronutrients from plant-based sources [25,26]. This raises additional concern about the possible increased risk of inadequate intakes of these nutrients in subjects consuming plant-based diets, including PBMAs, compared to those consuming meat [27]. This underscores the importance of considering the nutritional implications when making these dietary substitutions of animal foods with plant-based analogues [28].

Due to the growing presence of these plant-based foods on the market, an increasing number of studies have investigated the effects of substituting animal products with various types of plant-based alternatives on different markers of human health. However, to the best of our knowledge, the results of such studies have been systematically investigated for plant-based drinks but not for PBMAs or other plant-based alternatives [29]. Based on these premises, the aim of the present study is to systematically review evidence from human intervention studies investigating the impact of substituting AM with PBMAs in adults.

## 2. Materials and Methods

### 2.1. Search Strategy

A systematic literature search was conducted using two academic digital databases, PubMed^®^ and Scopus. The search was performed in October 2023 and updated in December 2023. The following syntax, which was adapted for each database, was used: (“plant-based” OR “vegeta*” OR mycoprotein OR “tofu” OR “soy”) AND (“meat” OR “chicken” OR “beef” OR “patties” OR “burger” OR “meatball*” OR “steak” OR “cutlet*” OR “pork”) AND (“alternative*” OR analog* OR substitut* OR meal) AND (“trial*” OR “study” OR “test”) AND (“human*” OR “adult*” OR consumer* OR “men” OR “women”).

**Figure 1:**
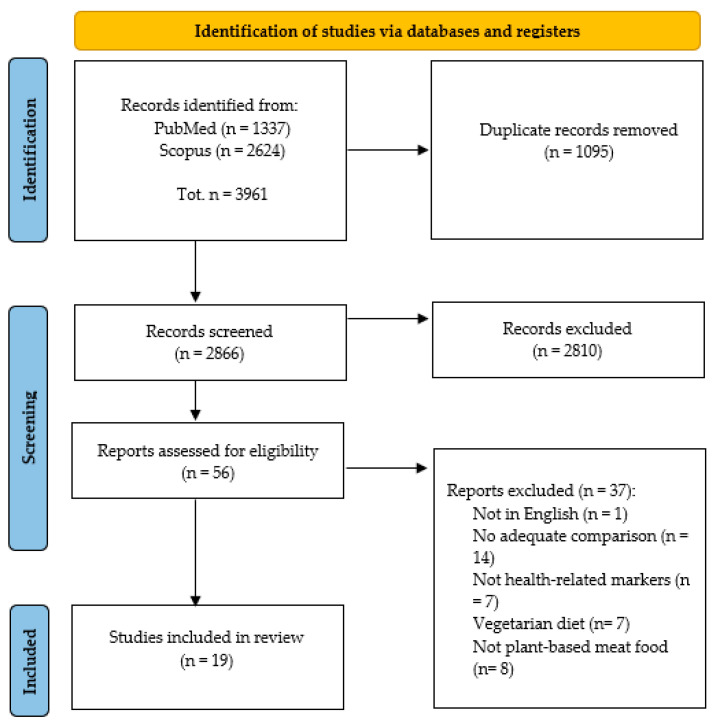
PRISMA flow diagram illustrating the literature search process and the application of selection criteria.

The search was limited to studies published from 2000 onwards. To ensure thoroughness and that all relevant articles were identified, references of the eligible articles were also consulted. The literature identification process was conducted in accordance with the PRISMA (Preferred Reporting Items for Systematic Reviews and Meta-Analyses) guidelines and is reported in Figure 1.

### 2.2. Study Selection

Articles were included if they reported dietary intervention studies that explored the impact of consuming meals with PBMAs (intervention) compared with meals composed of AM (control) on health-related parameters in humans. Exclusion criteria were applied based on age (≥18 years) but not on other participant characteristics (such as health status, body mass index (BMI), gender, and ethnicity). Another selection parameter was the language of publication, as only articles published in English were included.

The detailed list of eligibility criteria, developed following the PICOS (Population, Intervention, Comparison, Outcome, Study Design) format, is summarized in Table 1.

### 2.3. Data Collection

Two authors (F.C. and D.M.) independently screened studies and extracted data from eligible studies. Disagreements between reviewers were resolved through consultation with a third independent reviewer (C.D.B.) to reach a consensus. Data extracted included the year of publication, study design, location, sample size, characteristics of enrolled subjects, type and characteristics of tested PBMAs (intervention), characteristics of AM (control), study design, primary and secondary outcomes, and results.

### 2.4. Risk of Bias

An assessment of the risk of bias in individual studies and across the studies was carried out by two independent authors (L.C. and M.T.) (Supplementary Figures S1 and S2). The evaluation was carried out following the criteria of the Cochrane Handbook for Systematic Reviews of Interventions [30]. The analysis was structured into the following seven domains: (1) random sequence generation, (2) allocation concealment, (3) blinding of participants and personnel, (4) blinding of outcome assessment, (5) incomplete outcome data, (6) selective reporting, and (7) other bias. Each domain was judged as high risk, unclear risk, or low risk. Disagreements were resolved by consensus or by seeking consultation with another author (D.M.).

## 3. Results

### 3.1. Study Selection

A total of 3961 records were initially identified from the search on PubMed^®^ and Scopus. No additional results were identified through manual searches of reference lists. After 1095 duplicates were removed, the remaining 2866 studies were screened for their title and abstract, and 2810 were excluded. Among the 56 remaining studies, 37 records were excluded for not reporting results on any health markers, not being in English, or not providing a PBMA as the intervention.

At the end of the selection process, 19 trials were included in this review (Figure 1).

**Figure 2:**
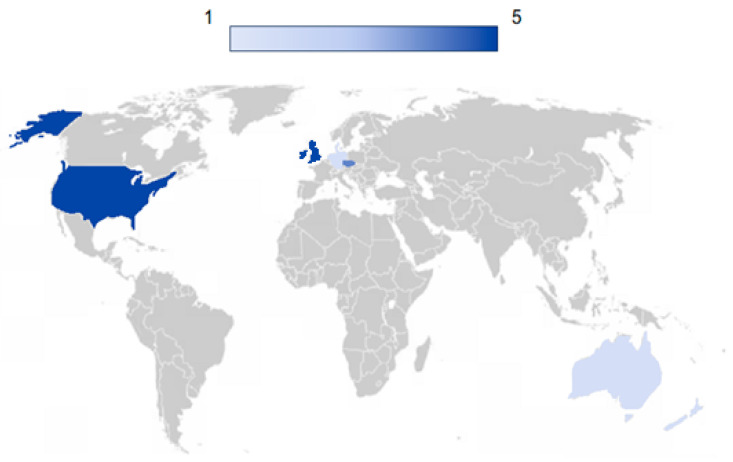
Geographical distribution of the studies included in this review. Blue indicates the number of studies performed in the different countries: the darker the blue, the higher the number of studies conducted.

The countries in which the 19 studies included in the review were conducted are reported in Figure 2. The largest number of included studies (*n* = 5) was carried out in the United States and the United Kingdom, followed by the Czech Republic (*n* = 4). Germany, Denmark, Australia, New Zealand, and the Netherlands contributed with a single study each.

### 3.2. Study Characteristics

All characteristics of the included studies are presented in Table 2, which reports data related to the year and country in which the study was performed, study design and duration (including any possible washout period), number of participants with their respective characteristics (i.e., age and BMI, health), PBMA and AM characteristics, outcomes, and results obtained.

The majority of the included studies described results from acute intervention trials (*n* = 11) in which a single dose of intervention and control meals were administered. In the other studies (*n* = 8), chronic effects were investigated, with an observation period ranging from one to eight weeks.

Out of the 19 articles analyzed, 16 were performed using a crossover design and 3 using a parallel design. A washout period was present in 15 studies, and it ranged from 3 days to 4 weeks.

Regarding the test meal, a hamburger (*n* = 11) was the most used meal in the intervention arm, followed by products prepared with mycoproteins in various forms (*n* = 5), minced vegetable meat (*n* = 1), and legume-based meatballs (*n* = 1). In one study, the PBMA type of product was not specified.

In 15 out of the 19 studies, the nutritional profile of the meals consumed was carefully outlined using tables in which the quantity of calories and macronutrients was indicated (in g or in %). On the contrary, the composition of the assigned meals was not reported in four studies [31,32,33,34]. However, one of them reported information related to the percentage of proteins contained in mycoprotein-based food and chicken-based food [32].

The control treatments mainly consisted of beef, followed by chicken and pork, always in the form of hamburgers, minced meat, meatballs, and sausages.

A total of 649 subjects were included within the 19 intervention studies considered in this review. The number of male subjects included in the analysis was more than double that of female subjects (*n* = 457 and *n* = 192, respectively). Eleven articles reported interventions in which only male adults were recruited, two studies recruited only females, and six studies recruited subjects from both sexes.

In one study, the effects of two different diets on calcium homeostasis were investigated, comparing menopausal with fertile women [34]. In another study, athletes were specifically enrolled to evaluate the impacts on sport performance [35].

In 13 studies, the target population consisted of healthy adults. Additionally, four studies examined the impacts of the two different diets on subjects with type 2 diabetes (T2D), six studies focused on overweight subjects (*n* = 6), and four studies focused on obese subjects (*n* = 4). The average BMI ranged from a minimum of 22.6 kg/m^2^ to a maximum of 34.5 kg/m^2^. One study reported only the BMI range without specifying the average [32]. The average age of the subjects ranged from a minimum of 24 years to a maximum of 50 years. Similarly, two studies reported only a range instead of the average age of the participants involved [32,33].

Supplementary Figures S1 and S2 report the risks of bias within individual studies and across the studies, respectively. Overall, the blinding of participants and personnel was identified as the highest risk of bias in the intervention studies.

### 3.3. Study Results

The main outcomes analyzed in the studies included in this review were satiety and postprandial fullness (*n* = 8); gastrointestinal and pancreatic hormones (*n* = 6); vascular functions and cardiovascular health (*n* = 4); intestinal microbiota health (*n* = 3); plasma amino acid response, muscle synthesis levels, and physical (sport) performance (*n* = 3); oxidative stress and inflammatory condition (*n* = 2); and calcium homeostasis (*n* = 1).

The results obtained from the comparison of the impacts of the consumption of meals composed of PBMA foods (intervention) and meals composed of AM (control) on health-related parameters are listed and described in Table 2. Given that there was only one study focused on calcium homeostasis that met the inclusion criteria [34], the findings from that study were mentioned. However, a discussion of the results was omitted.

#### 3.3.1. Effect on Markers of Satiety and Fullness

Among the 19 publications in the review, 8 compared the effects of consuming PBMAs and AM on satiety and fullness sensations. In all studies, the effect was investigated after a single meal administration. Furthermore, all studies were randomized controlled trials (RCTs) with a crossover design. Three studies considered satiety as the only research outcome, while the other five presented more than one outcome.

The first three interventions identified were crossover RCTs in which the authors investigated, on two separate days, the effect of consuming a vegetable burger (tofu burger) or a conventional meat burger (both 200 g) in 60 male subjects [36,37,38]. The subjects included 20 individuals with T2D, 20 individuals classified as overweight or obese, and 20 healthy individuals.

Regardless of the participants’ health status, a significant increase in satiety—assessed using a validated visual analogue scale (VAS)—was found following the consumption of a vegetable meal compared to an omnivorous meal in the interventions by Kahleova et al. and Klementova et al. [37,38]. Conversely, Malinska et al. objectively examined the effect on satiety, focusing on changes in hormone levels. A significant increase in leptin levels was observed in the groups of participants with diabetes and healthy participants after consuming a veggie burger compared to a conventional one. However, no effect was observed in the group of obese subjects [36]. Also, no differences were detected for ghrelin.

In the RCT by Muhlhausler et al., 24 healthy male adults consumed a meal consisting of pasta seasoned with either mycoprotein-based minced meat or beef-based minced meat on two different occasions [31]. The results showed that subjects consumed 586 kJ less when consuming pasta prepared with plant-based minced meat compared to pasta with minced beef at lunch. This energy reduction did not lead to a greater consumption during a buffet served 3 h later, to which they had ad libitum access. Furthermore, ghrelin and leptin concentrations did not differ between the two interventions.

A satiating effect following the consumption of plant-based meat was observed by Williamson et al. [32]. In the study, 42 overweight women consumed a preload of pasta seasoned with a mycoprotein-based meat substitute (intervention) or of isocaloric pasta enriched with chicken (control). The mycoprotein preload reduced food intake in the short term and in the following meal.

In the RCT by Kristensen et al., 43 male subjects consumed a breakfast consisting of legume meatballs with high (HP-legumes) or low (LP-legumes) protein content or veal and pork meatballs with high protein content (HP-meat) [39]. The results highlighted that satiety increased after the consumption of HP-legumes compared to HP-meat and LP-legumes, with an energy intake in the subsequent meal of 12% and 13% lower compared to HP-meat and LP-legumes, respectively.

Bottin et al. carried out two different interventions, with one involving 36 participants and the other involving 14 participants. The participants were either overweight or obese [40]. They consumed meals prepared with low (44 g), medium (88 g), or high (132 g) mycoprotein content or low (22 g), medium (44 g), and high (66 g) chicken content. Ad libitum energy intake was measured 3 h after the test meal, and gastric emptying was measured using the paracetamol method. A 10% reduction in energy intake was noted with high-mycoprotein-content meals compared to high-chicken-content ones.

The latest RCT acute study measured satiety in thirty healthy men consuming 220 g of PBMA Beyond Burger (BB) or 220 g of farm-raised meat. No significant differences were noted between groups in hunger and satiety scores [41].

#### 3.3.2. Effect on Markers of Oxidative Stress and Inflammation

The effect of substituting meat with PBMAs on oxidative stress and markers of inflammation was investigated in two studies.

Malinska et al. measured the onset of oxidative and dicarbonyl stress and the levels of inflammatory markers after a single meal in 60 subjects consuming 200 g of a PBMA (tofu burger) or 200 g of AM [36]. The results showed a decrease in the concentrations of oxidized glutathione (GSSG) and an increase in the activity of glutathione peroxidase (GPx) after the consumption of the PBMA compared to the AM. In subjects with obesity, consumption of the PBMA meal increased concentrations of reduced glutathione (GSH) and decreased concentrations of methylglyoxal. No differences were found in the secretion of tumor necrosis factor alpha (TNFα) and monocyte chemoattractant protein-1 (MCP-1).

In the work of Crimarco et al., 36 subjects consumed at least two portions per day of a PBMA (sausage, chicken, steak, veggie burger) or AM (sausage, chicken, beef steak, or burger) for 8 weeks. The results revealed no significant difference in inflammatory biomarkers between the two interventions, except for 4 out of the 92 biomarkers investigated, i.e., interleukin-7 (IL7), neurotrophin-3 (NT-3), FMS-like tyrosine kinase 3 ligand (FLT3L), and interleukin-22 receptor alpha 1 (IL22/RA1) [42].

#### 3.3.3. Effect on Markers of Vascular and Cardiovascular Health

Four studies analyzed the effect of PBMAs compared to AM products on vascular functions and/or on markers of cardiovascular health. The aim was to explore the impact of different products on the risk of atherosclerosis, hypertension, and ischemic heart disease. One study analyzed these markers after a single meal, while the remaining three studies tested the effects in the medium–long term.

In the single-meal study, Rudolph et al. evaluated the impact of consuming a veggie burger (203 g) platter compared to a beef burger platter on endothelium-dependent flow-mediated dilation (FMD) and markers of cardiovascular disease [43]. The effects were tested in 24 healthy subjects before the meal and 2 and 4 h post-consumption. The results showed that the decrease in FMD and plasma high-density lipoprotein (HDL) and the increase in brachial artery diameter and plasma triglycerides were dependent on time but not on the type of meal consumed, thus disproving the pre-established hypothesis.

Among the chronic studies, Crimarco et al., as mentioned before, investigated the chronic effect (8 weeks × 8 weeks without washout) of two food products in the form of burgers, sausages, and chicken strips in both plant-based and animal versions [44]. The study involved 36 healthy participants. It measured the level of trimethylamine-N-oxide (TMAO), which is an oxidation product released during the digestion of foods containing choline and carnitine, and other parameters related to cardiovascular risk such as blood pressure and lipid profile. The results showed that the levels of TMAO decreased only in the 18 subjects who, according to random assignment, had first received the animal meat-based intervention before the plant-based one. This was not the case in the second intervention arm in which the order was reversed and in which TMAO levels remained low even after switching to animal meat intervention. Furthermore, the value of body weight and low-density lipoprotein (LDL) cholesterol decreased after the plant-based phase compared to the animal meat-based one. No differences were found in blood pressure, HDL cholesterol, and triglyceride levels.

In the third study, authors compared the effect of substituting meat (i.e., chicken, ham, and beef) and fish (i.e., tuna and salmon) with PBMA products based on mycoproteins [33]. In total, 20 healthy adults were monitored for a period of one week of controlled diet. At the end of this period, a decrease in triglycerides, free cholesterol, LDL cholesterol, and HDL cholesterol was found in the intervention group compared to the control group.

Finally, Farsi et al. recently compared the effects of a PBMA meal formulated with mycoprotein-based products to a conventional meal made with processed red meat in 10 healthy subjects [45]. The results revealed that the PBMA phase caused a ~7% reduction in total cholesterol, a ~12% reduction in LDL cholesterol, and a decrease (even if not statistically significant) in systolic and diastolic blood pressure compared to the conventional meat phase (−4.11 mmHg and −2.71 mmHg relative to the meat diet).

#### 3.3.4. Effect on Pancreatic and Gastrointestinal Hormones

The impact of consuming plant-based versus animal-based meats on pancreatic or gastrointestinal hormone levels was investigated in six studies. In particular, studies focused on the release of insulin, peptide YY (PYY), amylin, and incretins such as glucagon-like peptide 1 (GLP-1) and glucose-dependent insulinotropic peptide (GIP). Out of these studies, four interventions evaluated the effect after a single meal, while the other two investigated the impact of chronic consumption.

Three crossover RCTs involved the administration of two types of meals: one meal consisting of a 200 g vegetarian burger and the other consisting of a 200 g beef burger, administered one week apart. Of the three RCTs, two [37,38] included the same group of 60 subjects (i.e., 20 healthy men, 20 overweight/obese subjects, 20 subjects with T2D), while the third study [46] included only the sub-group of 20 subjects diagnosed with T2D. In all three studies, an increase in GLP-1 was found in subjects with diabetes after consuming the plant-based meat compared to the animal-based one. In particular, Kahleova et al. [46] reported a 42% increase in GLP-1 secretion in subjects with diabetes and a 41% increase in secretion in overweight and obese subjects. Furthermore, a significant increase in insulin and amylin secretion, as well as PYY levels, emerged following the consumption of the PBMA meal compared to the control meal in all groups. In the third study, GIP decreased in subjects with diabetes after the consumption of a tofu burger in comparison to a conventional burger [46].

Bottin et al., as mentioned previously, recruited 36 volunteers and 14 volunteers for the first part and the second part of the study, respectively [40]. The participants were assigned to consume meals with low, medium, or high mycoprotein content or chicken content. The results showed that PYY, GLP-1, and blood glucose levels remained unchanged across the different interventions. However, the decrease in insulin concentration appeared to be significant after the intervention meals compared to the control meals. In the study by Crimarco et al. [44], the consumption of PBMA-based products for eight weeks did not reveal any significant variation in insulin secretion, regardless of the type of diet. Also, Coelho et al. reported similar results [33]. Specifically, no differences in insulin response and glucose levels were detected after one week of consuming mycoprotein products compared with the control diet in 20 healthy subjects.

#### 3.3.5. Effect on Gut Microbiota Composition

A total of three RCTs examined the long-term impacts of PBMA and AM products on gut microbiota composition. Crimarco et al. [44] found no difference in the composition of the intestinal microbiota after an 8-week consumption of a PBMA diet and an AM diet, in a group of 36 healthy subjects. Different results were obtained by Torribio-Mateas et al. [47]. Their study investigated the composition of the microbiota following a PBMA-based diet, which included burgers, sausages, minced meat, or meatballs made from rice, peas, or soy, in comparison to an omnivorous diet consisting of meat and other products of animal origin. The results revealed that the subjects who were assigned to the PBMA-based diet for a period of 4 weeks showed an increase in butyrate-metabolizing potential compared to the control group. In addition, an abundance of the taxa used in the fermentation of this molecule (Lachnospira, Faecalibacterium, Ruminococcaceae, and Oscillospira) was also found in the PBMA group, which also showed a lower quantity of bacteria belonging to the phylum Tenericutes.

Finally, Farsi et al. investigated the characteristics of the microbiota following a PBMA-based diet and an AM-based diet in 20 healthy men [45]. The participants consumed mycoprotein-based meals for a period of 2 weeks, followed by another two weeks of an ultra-processed red meat diet. Following the PBMA phase, the results showed an increase in the content of Proteobacteria, Verrucomicrobia, Akkermansia, and Roseburia, all of which are associated with inflammation suppression, and Lactobacillus, which has protective effects and improves intestinal function. Additionally, there was a decrease in the quantities of Faecalibacterium. An opposite trend in the bacteria listed above was seen following the AM consumption. This was accompanied by an increase in Oscillibacter bacteria, which are associated with weight gain, and a decrease in Ruminococcus and branched-chain fatty acids (BCFAs).

#### 3.3.6. Effect on Plasma Amino Acid Levels, Muscle Synthesis, and Physical Performance

Three studies focused on plasma amino acid levels and muscle synthesis after the consumption of PBMAs and AM. Two of these studies evaluated the effects after a single meal, while one evaluated the effects of a chronic intervention.

Kouw et al., in a parallel study involving 24 healthy male subjects, investigated the amino acid response in the plasma, the rates of muscle protein synthesis, and the signaling responses of muscle anabolism after a PBMA-based meal and a chicken-based meal [48]. The results showed that those consuming chicken had an increase in circulating essential amino acids (EAAs) and branched-chain amino acids (BCAAs), particularly leucine and methionine, compared to the PBMA group. In the PBMA group, an increase in lysine levels was found. No differences were noted in the quantities of non-essential amino acids (NEAAs) and in the rates of post-absorptive muscle protein synthesis following both diets.

In the second acute study, conducted by Pham et al., the plasma amino acid profiles in 29 healthy adults were evaluated after the consumption of 220 g of a PBMA (Beyond Burger) product and 220 g of AM consisting of lamb or beef [41]. The results showed a significant decrease in total amino acids, EAAs, NEAAs, and BCAAs at the plasma level following the consumption of PBMA products. This highlights a lower total protein bioavailability compared to AM consumption.

Finally, in a 12-week controlled, randomized, crossover study, Roberts et al. evaluated the effect of PBMA-based and meat-based diets on the physical performance of 22 athletes engaging in strength and muscular endurance exercises [35]. Among the 22 athletes, 11 were runners who had to carry out the 12 min Cooper test, while the remaining 11 were resistance trainers, who had to perform strength exercises with a machine and sequences of push-ups and pull-ups. Resistance and maximum VO2 levels were measured in the resistance trainers and runners, respectively. The results showed no significant differences in the Cooper test and VO2 maximum levels among the 11 runners. Similarly, in resistance trainers, no significant differences were observed in strength tests and the numbers of push-ups and pull-ups following the two different diets.

**Table 2:** Main characteristics and results of the included single-meal (a) and chronic (b) studies.

**a.** **Acute Intake (i.e., Single Meal)**						
**Reference and Country**	**Study Design**	**Study Population**	**Plant-Based Meat Analogue (PBMA)**	**Meat Intervention**	**Health Outcome**	**Health-Related Findings**
Kahleova et al., 2021 (Czech Republic) [37]	Postprandial, randomized, crossover study (1-week washout)	60 Caucasian men: 20 men with T2D Age: 48 ± 8.2 years BMI: 34.5 ± 3.4 kg/m^2^ 20 overweight/obese men Age: 43 ± 7.0 years BMI: 32.7 ± 3.9 kg/m^2^ 20 healthy men Age: 43 ± 7.1 years BMI: 23.8 ± 1.5 kg/m^2^	200 g of PB tofu burger (V-meal) 2154 kJ/514.9 kcal, 54.2 g carbohydrates, 19.9 g proteins, 22.8 g fats, 2.2 g SFA, 7.8 g fiber	200 g of conventional meat and cheeseburger (M-meal) 2149 kJ/513.6 kcal, 55 g carbohydrates, 20.5 g proteins, 22 g fats, 8.6 g SFA, 2.2 g fiber	Brain activity, gastrointestinal hormones, satiety	Thalamus perfusion: ↓ In men with T2D and O with M-meal vs. V-meal ↓ In H men with V-meal vs. M-meal Postprandial secretion of active GLP-1: ↑ 42% in men with T2D with V-meal vs. M-meal ↑ 41% in H men with V-meal vs. M-meal ↑ Satiety and insulin secretion following V-meal vs. M-meal in all men ↓ Palatability after V-meal vs. M-meal ⟷ Oxidative stress and inflammation in men with T2D and overweight/obese men after V-meal
Klementova et al., 2019 (Czech Republic) [38]	Postprandial, randomized, crossover study	60 Caucasian men: 20 men with T2D Age: 47.8 ± 8.2 years BMI: 34.5 ± 3.4 kg/m2 20 overweight/obese men Age: 43.0 ± 7.0 years BMI: 32.7 ± 3.9 kg/m^2^ 20 healthy men Age: 42.7 ± 7.1 years BMI: 23.8 ± 1.5 kg/m^2^	200 g of PB tofu burger (V-meal) 2154 kJ/514.9 kcal, 54.2 g carbohydrates, 19.9 g proteins, 22.8 g fats, 2.2 g SFA, 7.8 g fiber	200 g of conventional meat and cheeseburger (M-meal) 2149 kJ/513.6 kcal, 55 g carbohydrates, 20.5 g proteins, 22 g fats, 8.6 g SFA, 2.2 g fiber	Gastrointestinal hormones and satiety	↑ GLP-1 after V-meal vs. M-meal in T2D men and H men ↑ PYY after V-meal vs. M-meal in H men (18.9%) ↑ Amylin after V-meal vs. M-meal in all men (T2D: 15.7%; O: 11.5%; H: 13.8%) ↑ Satiety after V-meal vs. M-meal (T2D:9%; O:18.7%; H:25%)
Malinska et al., 2021 (Czech Republic) [36]	Postprandial, randomized, crossover study (1-week washout)	60 Caucasian men: 20 men with T2D Age: 47.8 ± 8.2 years BMI: 34.5 ± 11.9 kg/m^2^ 20 overweight/obese men Age: 43 ± 7.0 years BMI: 32.7 ± 3.9 kg/m^2^ 20 healthy men Age: 42.7 ± 7.1 years BMI: 23.8 ± 1.5 kg/m^2^	200 g of PB tofu burger (V-meal) 215.4 kJ/514.9 kcal, 54.2 g carbohydrates, 19.9 g proteins, 22.8 g fats, 2.2 g SFA, 7.8 g fiber	200 g of conventional meat and cheeseburger (M-meal) 2149 kJ/513.6 kcal, 55 g carbohydrates, 20.5 g proteins, 22 g fats, 8.6 g SFA, 2.2 g fiber	Postprandial oxidative and dicarbonyl stress, inflammatory markers, and appetite hormones	↓ T2D subjects after V-meal vs. M-meal: GSSG ↑ GPx ↑ Leptin ⟷ Methylglyoxal concentration ⟷ TNFα, MCP-1, or ghrelin Obese subjects after V-meal vs. M-meal: ↑ GSH ↓ Methylglyoxal ⟷ TNFα, MCP-1, or ghrelin Healthy subjects after V-meal vs. M-meal: ↑ Ascorbic acid ↑TNFα (but compared with T2D and O men is markedly lower) ↑Leptin (but compared with T2D and O men is markedly lower) ⟷ Ghrelin
Kahleova et al., 2019 (Czech Republic) [46]	Postprandial, randomized, crossover study (1-week washout)	20 participants with T2D Age: 47.8 ± 8.2 years BMI: 34.5 ± 3.4 kg/m^2^ HbA1c: 48.5 ± 8.1 mmol/mol at least three symptoms of the metabolic syndrome	200 g of PB tofu burger (V-meal) 215 4kJ/514.9 kcal, 54.2 g carbohydrates, 4 g sugars, 19.9 g proteins, 22.8 g fats, 2.2 g SFA, 7.8 g fiber	200 g of conventional meat and cheeseburger (M-meal) 2149 kJ/513.6 kcal, 55 g carbohydrates, 21 g sugars, 20.5 g proteins, 22 g fats, 8.6 g SFA, 2.2 g fiber	Postprandial secretion of incretins and insulin	⟷ Plasma GLU responses in V-meal and M-meal ↑ Immunoreactive insulin (30.5%), C-peptide (7.1%), amylin (15.7%) after V-meal vs. M-meal ↑ GLP-1 (19.2%) after V-meal vs. M-meal A positive relationship was found between Δ GLP-1 and Δ C-peptide and between Δ amylin and Δ C-peptide. ↓ GIP (−9.4%) after V-meal vs. M-meal ↑ Total insulin secretion and insulin secretion at a fixed GLU value 5 mmol/L after V-meal vs. M-meal ↑ Rate sensitivity ⟷ HOMA-IR
Rudolph et al., 2007 (Germany) [43]	Three days postprandial, observer-blinded, randomized, 3-way crossover trial (1-week washout)	24 healthy volunteers: (14 W, 10 M) Age: 32.0 ± 11.0 years BMI: 24.0 ± 5.0 kg/m^2^	203 g vegetarian burger: 493 kcal, 53 g carbohydrates, 10 g proteins, 25 g fats, 3 g SFA, 0.3 g trans fats, 8 mg vitamin C Test meal (meal 2): 203 g vegetarian burger, 152 g French fries, 20 mL ketchup, 500 mL carbonated lemon-flavored soda	211 g beef burger: 522 kcal, 44 g carbohydrates, 26 g proteins, 25 g all fats, 8.5 g SFA, 1.5 g trans fats, 1.5 mg vitamin C Test meal (meal 1): 211 g beef burger, 152 g French fries, 20 mL ketchup, 500 mL carbonated lemon-flavored soda	Acute effects on vascular function and cardiovascular biomarkers	Associated with time point but not type of meal: ↓ Flow-mediated endothelium-dependent dilatation (FMD) ↑ Diameter of the brachial artery ↑ Serum triacylglycerol concentration ↓ Serum HDL ⟷ Serum TC ↑ Plasma insulin after 2 h and 4 h ↑ Plasma GLU ⟷ ADMA (endogenous nitric oxide synthase inhibitor)
Muhlhausler et al., 2022 (Australia) [31]	Postprandial, single-blinded, randomized, cross-over trial (2 clinic appointments at least 1 week apart)	Healthy, adult males (*n* = 24) Age: 36.7 ± 2.0 years BMI: 24.0 ± 0.4 kg/m^2^	480 g PB mince composition: n.a. Test meal (785 kJ/100 g): pasta Bolognese dish (PB mince is 45% of the total cooked meal weight)	480 g beef mince composition: n.a. Test meal (788 kJ/100 g): pasta Bolognese dish (beef mince is 45% of the total cooked meal weight)	Satiety, fullness, and satisfaction	↓ 586 kJ of the pasta meal with PB vs. beef mince ⟷ Perception of hunger, satisfaction, and fullness with PB mince vs. beef mince ⟷ Plasma insulin and AUC for plasma insulin with PB mince vs. beef mince ⟷ Ghrelin concentration and AUC for ghrelin with PB mince vs. beef mince ↓ Plasma GLP-1 and AUC for plasma GLP-1 concentration (at 60–120 min) following PB meal vs. beef meal
Williamson et al., 2006 (Louisiana USA) [32]	Within-subject design (3-day test with at least 1 day of washout)	42 overweight adult females: Age range: 18–50 years BMI range: 25–29.9 kg/m^2^	MYC meal: 220 g of pasta with 44.3 g MYC (14 g protein/100 g) Composition MYC: n.a.	Chicken meal: 220 g of pasta with 20.2 g chicken (31 g protein/100 g) Composition chicken: n.a.	Satiety	MYC and tofu are associated with a stronger satiety effect in comparison to chicken during the lunch meal ↓ Food intake shortly after consuming the preload at lunch with MYC vs. chicken
Kouw et al., 2021 (the Netherlands) [48]	Parallel, double-blind, randomized, controlled trial	24 healthy, young active men: Age: 24 ± 5 years BMI: 22.9 ± 2.6 kg/m^2^	230 g of a baked lysine-enriched, plant-based meat substitute (Plant) Sources of lysine: wheat and chickpea flour and supplemented with 5% free lysine/100 g Composition per 100 g/per serving size: 559/1286 kJ, 7.4/39.9 g, proteins, 11.1/18.2 g, carbohydrates, 6.5/10.7 g fats	174 g of baked chicken breast (Chicken) Composition per 100 g/per serving size: 461/802 kJ, 23/39.9 g proteins, 0/0 g carbohydrates, 1.8/3.1 g fats	Plasma amino acid responses, muscle protein synthesis rates, and muscle anabolic signaling responses	↑ Plasma GLU concentration and plasma insulin concentration following Plant vs. Chicken ↑ Plasma EAA and BCAA concentrations following Chicken vs. Plant (Leu and Met) ↑ Postprandial plasma lys concentrations following Plant vs. Chicken ⟷ Postprandial NEAA concentrations or the sum of all amino acids when assessed over the entire 5 h postprandial period in Plant and Chicken ↑ Muscle protein synthesis rates compared with post-absorptive muscle protein synthesis rates in Plant and Chicken
Kristensen et al., 2016 (Denmark) [39]	Randomized, double-blind, placebo-controlled, three-way, cross-over meal test (washout period at least 2 weeks)	43 healthy, normal-weight, young men Age: 24.4 ± 4.8 years BMI: 23.0 ± 2.1 kg/m^2^	HP-Legumes: Fava bean (100 g) patties Nutritional composition of meal: 3552 kJ, 53% E carbohydrates, 19% E proteins, 28% E fats, 25 g fiber/100 g Serving weight: 591 g LP-Legumes: Fava bean (29 g) Patties Nutritional composition of meal: 3545 kJ energy, 62% E carbohydrates, 9% E proteins, 28%E fats, 10 g fiber/100 g Serving weight: 591 g	HP-Meat: veal and pork patties Nutritional composition of meal: 3546 kJ, 53% E carbohydrates, 19% E proteins, 28%E fats, 6 g fiber/100 g Serving weight: 591 g	Meal-induced appetite sensations	↓ Appetite score, hunger, prospective food consumption following HP-Legume vs. LP-Legume and HP-Meat ↑ Satiety after HP–Legume vs. HP-Meat A 12% and 13% lower energy intake was seen after HP-Legume compared to HP-Meat and LP-Legume, respectively
Kerstetter et al., 2006 (USA) [34]	Four 3-week cycles: 2-week adjustment period followed by a 4-day experimental period and 3 days of consuming food ad libitum	20 healthy women: 12 young Age: 29.2 ± 1.8 years BMI: 22.6 ± 0.8 kg/m^2^ 8 postmenopausal Age: 58.9 ± 1.6 years BMI: 25.1 ± 0.9 kg/m^2^	HP-soy-based (2.1 g/kg), LP-soy-based (0.7 g/kg) Products: Low-isoflavone soy protein isolate (Pro Fam 930) Harvest Burgers Recipe Crumbles Composition: n.a.	HP-meat-based (2.1 g/kg), LP-meat-based (0.7 g/kg). Composition: n.a.	Ca homeostasis	↑ At 24 h, NAE during the high-compared with low-protein intervention ↑ At 24 h, NAE during the meat compared with soy intervention ↑ 24 h urinary Ca with HP diets but not with the type of protein ↑ Serum concentrations of parathyroid hormone and calcitriol and urinary nephrogenous cAMP during the LP vs. HP intervention and during the soy vs. meat protein ⟷ Ca absorption with 4 diets
Bottin et al., 2016 (UK) [40]	Two randomized-controlled, single-blinded trials part A and part B(3–7-day washout period)	Part A 36 volunteers (17 W, 19 M) Age: 33 ± 14 years BMI 28·1 ± 2.3 kg/m^2^ Part B 14 volunteers (9 W, 5 M) Age: 37 ± 18 years BMI: 28.4 ± 2.5 kg/m^2^	MYC meal: low (44 g), medium (88 g), high (132 g) Composition (per serving): low: 418 kcal, 28 g carbohydrates, 24 g proteins, 9 g fats, 6 g fiber medium: 435 kcal, 26 g carbohydrates, 31 g proteins, 9 g fats, 31 g protein, 8 g fiber high: 445 kcal, 26 g carbohydrates, 37 g proteins, 8 g fats, 10 g fiber	Chicken meal: low (22 g), medium (44 g), high (66 g) chicken. Composition (per serving): low: 407 kcal, 30 g carbohydrates, 23 g proteins, 9 g fats, 4 g fiber medium: 418 kcal, 29 g carbohydrates, 30 g proteins, 8 g fats, 3 g fiber high: 424 kcal, 25 g carbohydrates, 40 g proteins, 9 g fats, 3 g fiber	Part A: the effect of three levels of MYC compared with chicken on appetite, acute and 24 h energy intake, glucose and insulin concentrations, and PYY and GLP-1 concentrations. Part B: the effect of the highest content of MYC used in part A compared with chicken on appetite, glucose and insulin concentrations, gastric emptying, energy expenditure, and substrate oxidation	↓ 10% energy intake (67 kcal) after MYC vs. chicken ↓ Insulin concentration after MYC vs. chicken ⟷ GLU, PYY, GLP-1, gastric emptying rate, and energy expenditure after MYC and chicken Following chicken intake, paracetamol-glucuronide was positively associated with fullness After MYC, creatinine and α-keto-β-methyl-N-valerate were inversely related to fullness, whereas the ketone body, β-hydroxybutyrate, was positively associated
Pham et al., 2022 (New Zealand) [41]	Postprandial, double-blinded randomized crossover trial (washout period of at least 1 week)	29 healthy men: Age: 28.0 ± 3.8 years BMI: 24.5 ± 2.7 kg/m^2^	220 g (raw) PBMA: Beyond Burger (Beyond Meat-BB): pea protein, canola oil and coconut oil Composition (cooked): 10.7 g proteins, 10.1 g fats, 18.3 g carbohydrates, 3.8 g sugars, 1.9 g fiber, 0.4 g sodium, 1.9 mg iron, 1.1 mg zinc, <0.5 mg cholesterol Test meal (approximately 470 g) is a burrito-style wrap containing BB + fresh and canned vegetables + tomato salsa + flour tortilla	220 g (raw) meat: Pasture-raised beef (Pasture) Grain-finished beef (Grain) Pasture-raised lamb (Lamb) Composition (cooked): Pasture: 10.3 g proteins, 11.1 g fats, 18.1 g carbohydrates, 3.5 g sugars,1.6 g fiber, 0.3 g sodium, <0.2 mg iron, 1.2 mg zinc, 27.9 mg cholesterol Grain: 11.2 g proteins, 6.7 g fats, 18.4 g carbohydrates, 4 g sugars,1.1 g fiber, 0.3 g sodium, <0.2 mg iron, 1.4 mg zinc, 26 mg cholesterol Lamb: 12.4 g proteins, 4.3 g fats, 19.1 g carbohydrates, 4 g sugars,1.7 g fiber, 0.3 g sodium, <0.2 mg iron, 1.2 mg zinc, 27.4 mg cholesterol Test meal (approximately 470 g) is a burrito-style wrap containing meat + fresh and canned vegetables + tomato salsa + flour tortilla	Primary outcome: postprandial digestive response after a single meal: appearance of AAs in plasma Secondary outcome: glucose and insulin, appetite assessment, and anthropometry	↓ The BB meal produced significantly lower plasma concentrations of total (TAAs), essential (EAAs: 7–28%), branched-chain (BCAAs), and NPAAs vs. meat, based on AUC ⟷ hunger, fullness, or cravings between meal groups
**b.** **Chronic Intake**						
Crimarco et al., 2020 (USA) [44]	Single-site, randomized, crossover trial (8 × 8-week and no washout period)	36 participants (24 W, 12 M) Age: 50.2 ± 13.8 years BMI: 27.9 ± 5.2 kg/m^2^	Plant products (≥2 servings/die): Burger (113 g) 250 kcal Beef crumbles (55 g) 90 kcal Breakfast sausage (65 g) 170 kcal Hot Italian sausage (76 g) 190 kcal Brat sausage (76 g) 190 kcal Grilled chicken strips (130 g) 130 kcal Lightly seasoned chicken strips (130 g) 130 kcal Nutritional composition range: 2–6 g carbohydrates, 12–22 g proteins, 2–18 g fats, <1–6 g SFA, 1–3 g fiber, 240–500 mg sodium	Animal products (≥2 servings/die): Burger (100 g) 293 kcal Ground beef (100 g) 293 kcal Good morning pork breakfast sausage (47 g) 110 kcal Hot Italian sausage (71 g) 170 kcal Pork bratwurst (57 g) 230 kcal Chicken breast (113 g) 140 kcal Nutritional composition range: 1–4 g carbohydrates, 7–26 g proteins, 3–25 g fats, 0.5–9 g SFA, 0 g fiber, 320–1402 mg sodium	Fasting serum TMAO, fasting insulin-like growth factor, lipids, GLU, insulin, blood pressure, weight, gut microbiota	TMAO: ⟷ *n* = 18 that received plant first (2.5 ± 0.4 PB; 3.0 ± 0.6 M) ↓ *n* = 18 that received animal first (2.9 ± 0.4 PB; 6.4 ± 1.5 M) ↓ LDL cholesterol after plant phase vs. animal phase (109.9 ± 4.5 mg/dL PB; 120.7 ± 4.5 mg/Dl M) ↓ Weight after plant phase vs. animal phase (78.7 ± 3.0 kg PB; 79.6 ± 3.0 kg M) ⟷ Fasting concentration of IGF-1, insulin, GLU, HDL cholesterol, TGs, blood pressure between plant phase and animal phase ⟷ Gut microbiota
Crimarco et al., 2022 (USA) [42]	Single-site, randomized, crossover trial (8 × 8-week and no washout period)	36 participants (24 W, 12 M) Age: 50.2 ± 13.8 years BMI: 27.9 ± 5.2 kg/m^2^	Plant products (≥2 servings/die): Burger (113 g) 250 kcal Beef crumbles (55 g) 90 kcal Breakfast sausage (65 g) 170 kcal Hot Italian sausage (76 g) 190 kcal Brat sausage (76 g) 190 kcal Grilled chicken strips (130 g) 130 kcal Lightly seasoned chicken strips (130 g) 130 kcal Nutritional composition range:2–6 g carbohydrates, 12–22 g proteins, 2–18 g fats, <1–6 g SFA, 1–3 g fiber, 240–500 mg sodium	Animal products (≥2 servings/die): Burger (100 g) 293 kcal Ground beef (100 g) 293 kcal Good morning pork breakfast sausage (47 g) 110 kcal Hot Italian sausage (71 g) 170 kcal Pork bratwurst (57 g) 230 kcal Chicken breast (113 g) 140 kcal Nutritional composition range: 1–4 g carbohydrates, 7–26 g proteins, 3–25 g fats, 0.5–9 g SFA, 0 g fiber, 320–1402 mg sodium	Biomarkers of inflammation	None of the change scores between the two diet phases are significantly different Only 4 of 92 inflammatory biomarkers reach statistical significance (IL7, NT-3, FLT3 L, IL22/RA1)
Coelho et al., 2021 (UK) [33]	Randomized, parallel-group trial (7-day dietary intervention)	20 healthy, recreationally active, young adults (12 W, 8 M) Range Age: 18–31 years Mean BMI: 23 kg/m^2^	215 ± 16 g/die MYC: Quorn chicken pieces, Quorn mince, Quorn fillets and Quorn roast chicken slices. Composition: n.a.	CTRL group: chicken, ham, beef, tuna and salmon Composition: n.a.	IS, glycemic control, and plasma lipoprotein composition	⟷ Blood GLU, serum insulin responses, IS, and 24 h glycemic profiles within and between groups ⟷ 171 of 224 metabonomic targets ⟷ 45 lipid concentration of different lipoprotein fractions in CTRL group ↓ 45 lipid concentration of different lipoprotein fractions in MYC group (7–27%) ↓ TC, free cholesterol, LDL-cholesterol, HDL-cholesterol, DHA, and *n*-3 fatty acids in MYC (14–19%) group vs. CTRL (3–11%)
Toribio-Mateas et al., 2021 (UK) [47]	Randomized, parallel, controlled study (4-week study period)	39 volunteers (20 W, 19 M) Age: 37.5 ± 8.9 years BMI: 23 ± 2.3 kg/m^2^	PBMA: Burger (pea and rice), Sausage (pea and rice), Mince (soy, pea and rice), Sausage Patty (pea), Meatballs (pea) Nutritional composition range: 199–234 kcal, 5.3–11.8 g carbohydrates, 14.4–19.1 g proteins, 10.9–15.9 g fats, 2.5–4.9 g fiber, 0.62–1.49 mg salt, 0 g cholesterol	No intervention. Subjects were requested to carry on consuming animal products including red meat, poultry, fish, eggs, and cheese daily	Changes to the gut microbiota	↑ Butyrate-production pathways in the PBMA groups but not in meat group ↑ Joint abundance of butyrate-producing taxa in PBMA vs. CTRL group ↓ Tenericutes phylum in the intervention group ↑ Tenericutes phylum in the control group
Farsi et al., 2023 (UK) [45]	Investigator-blind, randomized, crossover-controlled trial (2 × 2-week feeding blocks separated by a 4-week washout)	20 healthy male adults: Age: 30.4 ± 7.9 years BMI: 24.0 ± 2.9 kg/m^2^	240 g/day (uncooked weight) of MYC: Peppered steak, Sausages, Meat-free Ham Deli Slices, Gammon Steaks, Bacon Style Slices, Mince, and Hot Dogs Nutritional composition range: 269–514 kcal, 4.1–25.4 g carbohydrates, 24.2–39.8 g proteins, 4.8–37.7 g fats, 1.2–6.2 g SFA, 12.7–13.7 g fiber, 288–1536 mg sodium	240 g/day (uncooked weight) of red processed meat Beef Steak, Pork Sausages, Cold Cut Ham, Gammon Steak, Bacon Rashers, Beef Mince, and Hot Dogs Nutritional composition range: 200–540 kcal, 0–13.2 g carbohydrates, 28.8–52.2 g proteins, 6.7–45.6 fats, 3.4–19.7 g SFA, 0–4.2 g fiber, 134–1536 mg sodium	Markers of intestinal genotoxicity and gut health	↓ Fecal water genotoxicity after MYC vs. meat and basal level ↑ Fecal NOCs after meat phase ↓ Fecal NOCs after MYC ↓ Urinary p-cresol sulfate excretion in MYC phase ↓ 7-Ketodeoxycholic acid after meat phase ↑ Proteobacteria, Verrucomicrobia, Akkermansia, Roseburia, Lactobacillus in MYC phase ↓ Faecalibacterium in MYC phase ↓ Verrucomicrobia, Akkermansia, Roseburia, Ruminococcus in meat phase ↑ Faecalibacterium, Oscillibacter in meat phase ↓ BCFA following both diets but significant only after the meat phase
Roberts et al., 2022 (USA) [35]	Randomized crossover trial (4 × 4 × 4-week study, without washout periods)	22 athletes: 11 recreational runners: (5 W, 6 M) Age: 26.2 ± 4.6 years BMI (W): 22.4 ± 1.7 kg/m^2^ BMI (M): 23.3 ± 2.8 kg/m^2^ 11 resistance trainers: (5 W, 6 M) Age: 26.9 ± 4.3 years BMI (W): 21.5 ± 0.7 kg/m^2^ BMI (M): 24.7 ± 2.7 kg/m^2^	PBMA Impossible burger (4 oz): 240 kcal, 19 g proteins, 9 g carbohydrates, 14 g fats, 8 g SFA, 3 g fiber, 370 mg sodium Beyond Beef Ground (4 oz): 230 kcal, 20 g proteins, 7 g carbohydrates 14 g fats, 5 g SFA, 2 g fiber, 390 mg sodium Gardein Chick’n Strips (4 oz): 164 kcal, 18 g proteins, 6 g carbohydrates, 8 g fats, 1 g SFAs, 0 g fiber, 387 mg sodium	Animal burger (3 oz) 216 kcal, 21 g proteins, 0 g carbohydrates, 14 g fats, 5 g SFA, 0 g fiber, 57 mg sodium Pork (3 oz): 214 kcal, 23 g protein, 0 g carbohydrates, 13 g fats, 5 g SFA, 0 g fiber, 41 mg sodium Chicken Breast (3 oz): 147 kcal, 26 g proteins, 0 g carbohydrates, 4 g fats, 1 g SFA, 0 g fiber, 65 mg sodium	Primary outcome: endurance (Cooper 12 min timed run)—runners; muscular strength—resistance trainers Secondary outcome: VO2 max—runners; maximum push-up and pull-up test—resistance trainers	⟷ 12 min timed run following PBMA diet vs. meat diet (−2.9 m) (runners) ⟷ Machine composite strength, total kg and % following PMMA vs. meat (−0.7%) (resistance trainers) ⟷ VO2 max after PBMA diet vs. meat diet (runners) ⟷ Push-ups and pull-ups after meat diet vs. PBMA (resistance trainers)
Farsi et al., 2023 (UK) [45]	Investigator-blind, randomized, crossover-controlled trial (2 × 2-week feeding blocks separated by a 4-week washout)	20 healthy male adults: Age: 30.4 ± 7.92 years BMI: 24.0 ± 2.87 kg/m^2^	240 g/day (uncooked weight) of MYC: Peppered steak, Sausages, Meat-free Ham Deli Slices, Gammon Steaks, Bacon Style Slices, Mince, and Hot Dogs Nutritional composition range: 269–514 kcal, 4.1–25.4 g carbohydrates, 24.2–39.8 g proteins, 4.8–37.7 g fats, 1.2–6.2 g SFA, 12.7–13.7 g fiber, 288–1536 mg sodium	240 g/day (uncooked weight) of red processed meat: Beef Steak, Pork Sausages, Cold Cut Ham, Gammon Steak, Bacon Rashers, Beef Mince, and Hot Dogs Nutritional composition range: 200–540 kcal, 0–13.2 g carbohydrates, 28.8–52.2 g proteins, 6.7–45.6 fats, 3.4–19.7 g SFA, 0–4.2 g fiber, 134–1536 mg sodium	Biomarkers of cardiovascular risk	↓ 6.74% of total cholesterol after MYC phase from baseline ↓ 12.3% of LDL cholesterol after MYC phase from baseline ↓ Waist circumference for MYC vs. meat (−0.95 ± 0.42 cm) ⟷ Fasted TGs between MYC and meat ↓Mean SBP (−2.41 ± 1.89 mmHg) and DBP (−0.80 ± 1.23 mmHg) after MYC phase from baseline ↑ Urinary potassium (+126.12 ± 50.30 mmol/L) and nitrate (+2.12 ± 0.90 mmol/L) after MYC vs. meat

Health-related findings report only significant results. Data are presented as means ± standard deviation. Abbreviations: ADMA: asymmetric dimethylarginine; AUC: area under the curve; BB: Beyond Burger; BCAA: branched-chain amino acid; BCFA: branched-chain fatty acid; BMI: body mass index; Ca: calcium; CTRL: control; DBP: diastolic blood pressure; EAA: essential amino acid; FLT3L: FMS-like tyrosine kinase 3 ligand; FMD: brachial-artery-flow-mediated dilation; GIP: glucose-dependent insulinotropic polypeptide; GLP-1: glucagon-like peptide-1; GLU: glucose; GPx: glutathione peroxidase; GSH: reduced glutathione; GSSG: oxidized glutathione; HDL: high-density lipoprotein; HP: high protein; IL22/RA1: Interleukin-22 receptor, alpha 1; IL7: Interleukin-7; IS: insulin sensitivity; LDL: low-density lipoprotein; Leu: leucine; LP: low protein; Lys: lysine; M: men; M-meal: meat meal; MCP-1: Monocyte Chemoattractant Protein-1; Met: methionine; MYC: mycoprotein; n.a.: not available; NAE: net acid excretion; NEAA: nonessential amino acid; NOC: N-nitroso compound; NPAA: non-proteogenic amino acid; NT-3: Neurotrophin-3; PB: plant-based; PBMA: plant-based meat analogue; PYY: Peptide YY; SBP: systolic blood pressure; SFA: saturated fatty acid. T2D: type 2 diabetes; TAA: total amino acid; TC: total cholesterol; TG: triglyceride; TNF-α: tumor necrosis factor; TMAO: trimethylamine-N-oxide; V-meal: vegetarian meal; W: women; ↑: significant increase; ⟷: no significant changes; ↓: significant decrease.

## 4. Discussion

The shift towards a diet rich in plant-based foods instead of animal-based ones is widely recommended due to growing concerns about the impact of diet on human, animal, and planetary health [5]. Consequently, in recent years, plant-based meat substitutes that mimic their traditional counterparts in appearance, taste, and consistency have recorded an increase in production and consumption. However, this rapid increase in consumer demand has raised questions regarding potential health concerns such as allergens, toxicity, and nutritional adequacy. The current review summarizes the 19 studies that assessed the health effects of replacing AM with PBMAs.

One of the most studied and interesting outcomes analyzed was the satiating effect associated with the consumption of legume- and cereal-based PBMA products compared to the consumption of AM. The effect was generally coupled with a lower energy intake during the same and the following meal. These results can be attributed to the higher fiber content, which is absent or present in negligible quantities in AM, together with a beneficial modulation of gastrointestinal hormones [22]. It is important to underline that EFSA does not consider the measurement of satiety sensations alone to be sufficient to support a health claim [49]. However, the measurement of postprandial satiety, along with other factors, can help provide an understanding of how a food or component may positively impact energy balance and control body weight. Thus, despite the need for further evidence, PBMA products might be considered favorable in a balanced diet aimed at preventing or reducing weight gain [50]. In this regard, Medawar et al. have noted a decrease in body weight following the consumption of plant-based products in a systematic review investigating the effects of a plant-based diet compared with a conventional diet [51]. Interestingly, this result was observed despite the same amount of energy being consumed with both diets. Increasing the intake of fiber from legumes and cereals has also been identified as an important factor that could induce beneficial metabolic effects. These effects may include the fermentation of carbohydrates, the regulation of appetite driven by intestinal hormones, the slowing of digestion, and an improved control of lipids.

The potential benefits associated with the higher fiber content in PBMAs compared to AM can also be related to the maintenance of a healthy intestinal microbiota. It is known that diets have a major role in shaping the composition and function of gut microbiota [52]. In this context, plant-based diets have been shown to exert beneficial effects when compared to conventional diets, which makes it reasonable to expect a positive modulation of gut microbiota when a PBMA is consumed in place of AM [53]. However, while two studies found a greater production of both short-chain fatty acids (e.g., butyrate) and associated taxa after replacing AM with corresponding PBMAs [45,47], one study reported no positive modulation in the composition of the intestinal microbiota. This suggests the need for further investigations aimed at exploring this potential beneficial effect [44].

Regarding the impact on hormones, conflicting results were observed. Gastrointestinal hormones are greatly involved in the regulation of glucose metabolism, energy homeostasis, satiety, and weight control. Thus, they play a pivotal role in the prevention of diseases like T2D. Numerous observational studies indicated that an increased consumption of red and processed meat is positively correlated with the risk of T2D and gestational diabetes. In particular, it has been shown that a daily addition of 30 g of processed red meat to the diet is associated with a relative risk of 1.5 of gestational diabetes, suggesting a greater risk as consumption quantities increase [54,55]. This may be due to the content of saturated fats and other compounds within meat that may promote oxidative stress and thus increase the risk of T2D and cardiovascular diseases. Conversely, there is convincing evidence that the intake of plant-based foods is associated with a lower risk of T2D [56,57]. However, out of the six studies included in this review that analyzed gastrointestinal and pancreatic hormones, only three studies noted improvements in hormone release [37,38,46]. The remaining three did not find any significant changes in insulin sensitivity, blood glucose, and gastrointestinal hormones [33,40,44]. Due to the limited number and the heterogeneity of results, no definitive conclusions can be drawn on this matter.

Similarly, studies analyzing the inflammatory response and oxidative stress found limited evidence. Indeed, only two trials specifically addressed and compared the effects of PBMAs and AM products on inflammatory biomarkers [36,42]. Diets rich in highly processed foods, saturated fatty acids, and simple sugars demonstrate the ability to increase inflammatory factors (IL-6, TNF-α, IL-1 β). In contrast, nutritional approaches characterized by minimally processed foods, an abundance of antioxidants, and a low glycemic index can reduce these inflammatory factors and the risks associated with them. Therefore, the use of legumes and vegetables in the preparation of PBMAs could play a role in dampening inflammation and promoting protection against related pathologies [58,59,60,61]. However, the two studies included in this review reported conflicting outcomes, likely due to the analysis of systemic rather than local inflammatory markers, thus not allowing conclusions to be drawn.

A small number of publications have analyzed cardiovascular health status related to the replacement of AM with PBMAs. Within the publications, the most investigated markers were plasma lipids, blood-flow-mediated vasodilation, TMAO concentrations, and blood pressure levels. Observational studies indicated that consumption of red and processed meat, which is generally higher in saturated fat and cholesterol, is linked to an increased risk of heart disease, raising concerns about cardiovascular health [57,62]. In this review, it was found that current research mainly focuses on comparing the consumption of mycoprotein products with AM. The results of the included studies suggest a potential improvement in lipoprotein, cholesterol, and triglyceride levels following PBMA interventions. Furthermore, favorable reductions are observed in TMAO levels when switching from a meat-rich diet to a plant-based one, consistent with previous findings [63]. However, the underlying mechanisms of such results are not yet fully understood and require further investigation.

Finally, a limited number of studies included in the review examined the impacts of consuming plant-based meat analogues compared to traditional meat on muscle protein synthesis levels and physical performance. Proteins of plant origin are often considered less effective in promoting muscle anabolism than proteins of animal origin, due to their lower protein quality and biological value (i.e., lower content of indispensable amino acids) and their bioavailability. Kouw et al. highlighted that the enrichment of PBMAs with limiting amino acids could represent a strategy to improve their effectiveness [48]. The authors suggested that special attention should be given to amino acids such as lysine and methionine, or BCAAs, for products targeted to specific dietary needs.

In this context, regarding physical performance, two studies included in the review suggested that a fortified formulation of PBMAs could be an effective solution to maintain physical performance when shifting towards more plant-based diets, despite the reduced bioavailability of total and essential amino acids seen with the consumption of PBMAs compared to AM [41,48]. Furthermore, the only study that analyzed physical performance in runners and amateur trainers did not demonstrate different rates of muscle synthesis and muscle strength following a vegetarian and omnivorous diet [35]. These results, in accordance with previous findings, seem to disprove the false belief that physical performance is negatively influenced by a vegetarian diet due to presumed inferior muscle development and inadequate energy intake during the recovery period [64]. However, due to the limited number of studies in this regard, further investigations should be carried out.

The present review has some strengths and limitations worth noting. While it was a systematic review, a limitation is that only 19 studies were included. This suggests that, despite the growing interest in this topic, more studies are needed to better elucidate the impact of PMBA on human health. This is particularly important for markers that have been the object of few studies, such as markers of calcium homeostasis, which have been considered in only one study so far. Moreover, most of the studies included in the review were performed in North American and European countries. These findings suggest the need to perform future investigations also in Asian, South American, and African countries to clarify the impact of geographical location on the outcomes under evaluation.

Among its strengths, to the best of our knowledge, this is the first systematic review focusing on the impact of substituting AM with PBMAs and considering a wide range of markers of human health.

In conclusion, this systematic review examined the impact of PBMA consumption on various health outcomes. The results showed that PBMA consumption improved satiety but resulted in lower protein bioavailability and smaller changes in plasma essential amino acids in comparison to AM. However, no clear effects on muscle protein synthesis and physical performance were reported, and there were conflicting results regarding hormonal activity, oxidative stress, inflammation, vascular function, and microbiota composition. The findings of these studies should be considered in light of some limitations. We noted significant heterogeneity among the studies we reviewed, including in their design, outcomes, and findings and the populations they studied. Furthermore, most of the studies that were assessed were acute interventions, with little consideration given to the long-term effects of substituting AM with PBMAs, and there was limited evaluation of the nutritional adequacy of both the specific PBMAs and the overall diet. Thus, all these points underscore the urgent need for future research to investigate the long-term effects of PBMAs on health outcomes and to ensure comprehensive nutrition assessments. This future research could contribute to a clearer understanding of how PBMA consumption affects health and clarify the possibility of including these products in sustainable and healthy diets for different populations.

## Figures and Tables

**Table 1:** PICOS design for the formulation of eligibility criteria.

Parameter	Inclusion Criteria
Population	Age > 18 years
Intervention	Dietary intervention evaluating the effect of a PBMA
Comparison	Animal-based meat
Outcome	Health and disease markers
Study design	Human intervention studies

PBMA: plant-based meat analogue.

## Data Availability

The data presented in this study are available on request from the corresponding author.

## Supplementary Materials

The following supporting information can be downloaded at https://www.mdpi.com/article/10.3390/nu16152498/s1: Figure S1: Risk of bias for each item assessed in each of the included studies; Figure S2: Risk of bias for each item assessed, presented as a percentage across all included studies combined.
