# Supplementary material for: Impact of Substituting Meats with Plant-Based Analogues on Health-Related Markers: A Systematic Review of Human Intervention Studies
Source: Nutrients. 2024 Jul 31;16(15):2498. doi: 10.3390/nu16152498 (PMC11314210; doi:10.3390/nu16152498)

Supplementary Materials

Figure S1. Risk of bias for each item assessed in each of the included studies.

	D1	D2	D3	D4	D5	D6	D7
Bottin et al., 2016	+	–	x	–	x	x	–
Coelho et al., 2021	+	–	+	–	–	+	–
Crimarco et al., 2020	–	+	x	+	+	+	–
Crimarco et al., 2022	+	+	x	+	+	+	–
Farsi et al., 2023a	+	+	x	+	+	+	–
Farsi et al., 2023b	+	+	x	+	–	–	–
Kahleova et al., 2019	+	+	x	+	+	+	–
Kahleova et al., 2021	+	+	x	+	x	+	–
Kerstetter et al., 2006	–	–	–	+	x	+	–
Klementova et al., 2019	+	+	x	+	–	–	–
Kouw et al., 2021	+	+	+	+	+	+	–
Kristensen et al., 2016	+	+	x	–	+	+	–
Malinska et al., 2021	+	+	x	+	–	+	–
Muhlhausler et al., 2022	+	+	+	+	+	+	–
Pham et al., 2022	+	+	+	+	+	+	–
Roberts et al., 2022	+	+	x	–	+	+	–
Rudolph et al., 2007	–	–	x	+	+	+	–
Toribio-Mateas et al., 2021	–	+	x	+	+	x	–
Williamson et al., 2006	–	–	–	–	x	+	–

Figure S2. Risk of bias for each item assessed, presented as a percentage across all included studies combined.

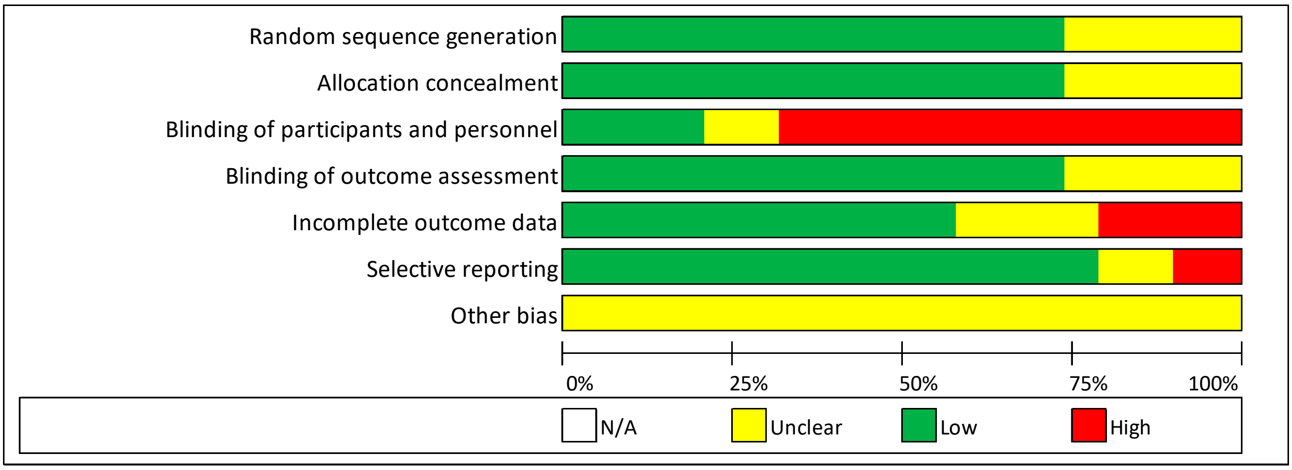

Supplement: Supplementary file 1 [file nutrients-16-02498-s001.zip › nutrients-3104866-supplementary.pdf]
